# Complement Factor D Is a Novel Biomarker and Putative Therapeutic Target in Cutaneous Squamous Cell Carcinoma

**DOI:** 10.3390/cancers14020305

**Published:** 2022-01-08

**Authors:** Pegah Rahmati Nezhad, Pilvi Riihilä, Jaakko S. Knuutila, Kristina Viiklepp, Sirkku Peltonen, Markku Kallajoki, Seppo Meri, Liisa Nissinen, Veli-Matti Kähäri

**Affiliations:** 1 Department of Dermatology, University of Turku and Turku University Hospital, Hämeentie 11 TE6, FI-20520 Turku, Finland; perane@utu.fi (P.R.N.); pimati@utu.fi (P.R.); jsknuu@utu.fi (J.S.K.); krivii@utu.fi (K.V.); sipelto@utu.fi (S.P.); liinis@utu.fi (L.N.); 2 FICAN West Cancer Centre, Research Laboratory, University of Turku and Turku University Hospital, Kiinamyllynkatu 10, FI-20520 Turku, Finland; 3 Department of Pathology, University of Turku and Turku University Hospital, Kiinamyllynkatu 10, FI-20520 Turku, Finland; markku.kallajoki@tyks.fi; 4 Department of Bacteriology and Immunology, The Translational Immunology Research Program, University of Helsinki, FI-00014 Helsinki, Finland; seppo.meri@helsinki.fi

**Keywords:** complement, cutaneous squamous cell carcinoma, skin, progression, factor D, adipsin, Danicopan, ACH-4471, biomarker, therapeutic target

## Abstract

**Simple Summary:**

The incidence of the most common metastatic skin malignancy, cutaneous squamous cell carcinoma (cSCC), is growing worldwide, and the prognosis of the metastatic disease is poor. Presently, there are no biomarkers or therapeutic targets for high-risk cSCCs. Recent studies have demonstrated the essential role of autocrine complement synthesis in the progression of cSCC. Here, we have evaluated the role of complement Factor D (FD), the rate-limiting enzyme of the alternative complement pathway, in cSCC development. The results identify FD as a novel biomarker and putative therapeutic target for cSCC and propose the small-molecule FD inhibitor Danicopan as a highly specific drug candidate in the therapy of advanced cSCC. It is expected that the discovery of complement-associated molecular markers for cSCC progression would improve diagnosis, classification, prognostication, and targeted therapy of cSCC and its precursors in the future.

**Abstract:**

Cutaneous squamous cell carcinoma (cSCC) is the most prevalent metastatic skin cancer. Previous studies have demonstrated the autocrine role of complement components in cSCC progression. We have investigated factor D (FD), the key enzyme of the alternative complement pathway, in the development of cSCC. RT-qPCR analysis of cSCC cell lines and normal human epidermal keratinocytes (NHEKs) demonstrated significant up-regulation of FD mRNA in cSCC cells compared to NHEKs. Western blot analysis also showed more abundant FD production by cSCC cell lines. Significantly higher FD mRNA levels were noted in cSCC tumors than in normal skin. Strong tumor cell-associated FD immunolabeling was detected in the invasive margin of human cSCC xenografts. More intense tumor cell-specific immunostaining for FD was seen in the tumor edge in primary and metastatic cSCCs, in metastases, and in recessive dystrophic epidermolysis bullosa-associated cSCCs, compared with cSCC in situ, actinic keratosis and normal skin. FD production by cSCC cells was dependent on p38 mitogen-activated protein kinase activity, and it was induced by interferon-γ and interleukin-1β. Blocking FD activity by Danicopan inhibited activation of extracellular signal-regulated kinase 1/2 and attenuated proliferation of cSCC cells. These results identify FD as a novel putative biomarker and therapeutic target for cSCC progression.

## 1. Introduction

The two keratinocyte-derived carcinomas, basal cell carcinoma (BCC) and cutaneous squamous cell carcinoma (cSCC), are the most common cancers globally [1,2]. cSCC has been reported to account for 20% of keratinocyte carcinomas, and its worldwide incidence is growing [3,4,5,6,7,8]. There is a 3–7% potential for metastasis of invasive primary cSCC, and patients with the metastatic disease show a poor prognosis [3,4,5,6,7,8]. cSCC develops from premalignant lesion actinic keratosis (AK) to cSCC in situ (cSCCIS) (Bowen’s disease) and eventually to invasive cSCC, which can progress to metastatic cSCC [9]. Ultraviolet (UV) irradiation is the leading predisposing factor for cSCC, followed by chronic cutaneous ulceration and immunosuppression [1,2]. Moreover, chronic local inflammation promotes the progression of cutaneous neoplasia [9,10,11].

The complement system connects innate and adaptive immunity and is considered as a significant effector arm of host immune defense. It is composed of three individual pathways: classical, lectin and alternative pathways [11,12,13,14,15]. Sequential activation of these cascades results in the conversion of central complement component C3 to C3a and C3b activation products. The latter is an opsonin that promotes the phagocytosis of pathogens by leukocytes. C3b is part of the alternative pathway (AP) C3 convertase C3bBb generating more C3b molecules in the so-called amplification cycle. It also initiates the establishment of C5 convertases (C4bC2bC3b or C3b_2_Bb), which, in turn, activate C5 to C5a and C5b. C5a and C3a are anaphylatoxins that activate an inflammatory response, and C5b initiates activation of the terminal pathway of complement, which leads to the formation of membrane attack complex (MAC) capable of target cell lysis [11,12,13,14,15].

The alternative pathway is continuously activated at a low rate by the spontaneous hydrolysis of C3 to yield C3 (H_2_O). C3 (H_2_O) binds to complement factor B (FB) to create the C3 (H_2_O) B complex. Complement factor D (FD; also known as adipsin) is a pivotal activator of AP, and it is strictly specific for FB [11,12,13,14,15]. This key rate-limiting enzyme of AP is a 24 kDa serine protease that cleaves FB in C3 (H_2_O) B and generates the fluid phase C3 convertase (C3 (H_2_O) Bb). This initial C3 convertase then cleaves new C3 molecules into C3a and C3b, and the attachment of FB to C3b forms the intermediate C3bB complex, which subsequently, after cleavage of FB by FD, becomes the active C3 convertase (C3bBb) [11,12,13,14,15]. The active C3 convertase, stabilized by properdin (P), cleaves additional C3 molecules into C3a and C3b, thus self-amplifying its own activation through a positive feedback loop. FD circulates in the latent self-inhibited form in plasma, and its activation is dependent on conformational transformation upon association with a specific substrate, i.e., C3 (H_2_O) B or C3bB [11,12,13,14,15]. In addition, latent proFD is activated to FD by MASP3 [16]. FD is mainly synthesized by adipocytes, hence the alias name adipsin [17]. FD is also produced by monocytes, macrophages and liver cells [18]. The plasma concentration of FD (1–2 μg/mL) is the lowest among complement components, and its excretion route is renal [19].

The cancer-promoting properties of tumor cell-derived complement components have been recently demonstrated, and they have been shown to promote cancer progression independently of local and systemic complement activation [11,20,21,22,23,24]. Our previous studies have revealed significant upregulation of four complement system activators (FB and C3 in AP; C1r and C1s in the classical pathway) plus two complement inhibitors, complement factor I (FI) and complement factor H (FH), in cSCC cell lines in vitro as well as in tumor cells in cSCC in vivo, when compared with normal human epidermal keratinocytes (NHEKs) and normal skin, respectively [25,26,27,28,29,30].

Here, we have examined the role of FD in cSCC development and progression. The results demonstrated that FD was markedly expressed by cSCC cells in culture and in vivo. The FD expression level in vivo was markedly higher in non-metastatic cSCC, metastatic cSCC, cSCC metastasis and recessive dystrophic epidermolysis bullosa-associated cSCC (RDEBSCC), compared to normal skin, premalignant lesion, actinic keratosis (AK) and cSCC in situ (cSCCIS). The strongest expression of FD was localized to the invasive edges of the tumors, whereas the weak FD staining pattern in normal skin, AK and cSCCIS showed a diffuse homogenous distribution. Blocking the activity of FD with danicopan inhibited the proliferation of cSCC cells. These findings identify FD as a novel potential diagnostic biomarker for the progression of cSCC and a therapeutic target in advanced and metastatic cSCC.

## 2. Materials and Methods

### 2.1. Cell Cultures

Human cSCC cell lines were established from surgically excised cSCCs [31]. cSCC cell lines UT-SCC-12A, UT-SCC-91, UT-SCC-105, UT-SCC-111 and UT-SCC-118 were initiated from primary cSCCs and cSCC cell lines UT-SCC-7, UT-SCC-59A and UT-SCC-115 from metastatic cSCCs. Authentication of the cell lines was performed by short tandem repeat (STR) DNA profiling [32]. NHEK cultures (*n* = 10) were established from skin obtained from mammoplasty procedures carried out for esthetic and functional purposes in Turku University Hospital [31]. Primary human epidermal keratinocytes (NHEK-PC) were obtained from PromoCell (Heidelberg, Germany). The cell culture protocol was according to the previously published studies [31,32,33]. To evaluate the impact of inflammatory cytokines, cSCC cell cultures were maintained in serum-free Dulbecco’s modified Eagle’s medium (DMEM) for 24 h and subsequently treated with interferon-γ (IFN-γ) (100 U/mL) (Promega, Madison, WI, USA) or interleukin-1β (IL-1β) (10 ng/mL) (Calbiochem, San Diego, CA, USA) for 24 h [25]. To examine the role of MAPK signaling in FD expression, cSCC cells were treated with MEK1/2 inhibitor PD98059 (30 μM), or with p38α/p38β inhibitor SB203580 (10 μM), or with p38α/p38β/p38γ/p38δ inhibitor BIRB796 (10 μM) (all from Calbiochem) for 24 h.

HaCaT, a spontaneously immortalized non-tumorigenic human keratinocyte-derived cell line [34] and three Ha-ras-transformed tumorigenic HaCaT cell lines (A5, II-4 and RT3) [35] were kindly provided by Dr. Norbert Fusenig (Deutsche Krebsforschungszentrum, Heidelberg, Germany). A5 cells form benign, II-4 cells low-grade malignant and RT3 cells high-grade malignant tumors in vivo in nude mice [36].

### 2.2. Tissue RNA

Normal human skin specimens (*n* = 10) were acquired from patients who underwent mammoplasty surgery in Turku University Hospital or the upper arm of healthy volunteers. Primary cSCC specimens (*n* = 6) were obtained from surgically excised tumors in Turku University Hospital [31]. Total RNA was isolated from the tissue samples, as previously described [37].

### 2.3. Real-Time Quantitative PCR (RT-qPCR)

cDNA synthesis after total RNA extraction was carried out in accordance with the previous protocols [25]. qPCR analysis was then performed by the QuantStudio 12K Flex (Thermo Fisher Scientific, Waltham, MA, USA), utilizing specific primers and probes for FD and β-Actin. The following primers and probes were used for FD:

FD forward primer 5′- GGGTCACCCAAGCAACAAAG -3′

FD reverse primer 5′- CGTGGCCCATGCTGATCTC -3′

FD probe 5′-Fam-TCCCGAGCAATGAAGTCATCCAC-Tamra-3′

Primers and probes for β-Actin have been described previously [25]. The threshold cycle values (Ct) were below 5% of the mean, and samples were analyzed in triplicates. β-Actin mRNA levels were used as the internal control.

### 2.4. Western Blot Analysis

FD protein levels in conditioned media of NHEKs, untreated and DMSO/PD/SB/BIRB-treated cSCC cell lines, HaCaT and tumorigenic Ha-ras-transformed HaCaT cells were determined with Western blotting under non-reducing conditions using a specific polyclonal rabbit anti-FD antibody (SAB1301593, Sigma-Aldrich Chemie GmbH, Steinheim, Germany; 1:270). Lysates of DMSO, PD98059, SB203580, BIRB796 and FD inhibitor (danicopan; ACH-4471) treated cSCC cells were analyzed with antibodies specific for phosphorylated ERK1/2 (p-ERK1/2) and total ERK1/2 (9101 and 9102, Cell Signaling Technology, Beverly, MA, USA) or p-CREB (9191, Cell Signaling Technology) under reducing conditions, dilution 1:1000. In total cell lysate samples, equal protein loading was validated with β-Actin antibody (AC-15, A-1978, Sigma-Aldrich, St. Louis, MO, USA; dilution: 1:4000), and in cSCC cell medium samples with anti-TIMP-1 antibody (MAB3300, Millipore; dilution: 1:1000), both under reducing conditions.

### 2.5. Tissue Samples and Immunohistochemistry

Altogether, 425 formalin-fixed paraffin-embedded tissue samples were collected from the archives of the Department of Pathology, Turku University Hospital and of the Auria Biobank, Turku University Hospital and the University of Turku. These tissue samples comprised of sporadic UV-induced non-metastatic primary cSCC (*n* = 140), sporadic UV-induced metastatic primary cSCC (*n* = 70), cSCC metastases (*n* = 9), cSCCIS (Bowen’s disease; *n* = 61), AK (*n* = 65) and normal skin (*n* = 80) [8]. Additional tissue samples included RDEBSCCs (*n* = 16) [38,39]. Original tissue samples were assembled to tissue microarray (TMA) blocks, as described earlier [40]. The sections were stained with immunohistochemistry (IHC) using rabbit polyclonal anti-FD (SAB1301593, Sigma-Aldrich Chemie GmbH, Steinheim, Germany) in Histocore Facility of the Institute of Biomedicine, University of Turku, as previously described [25,26,27,28]. The slides were counterstained with hematoxylin. The stained slides were scanned using a Pannoramic 250 Slide Scanner or a Pannoramic 1000 Slide Scanner (3DHistech, Budapest, Hungary). The immunostaining of FD was scored based on the intensity of cytoplasmic staining as negative (−), weak (+), moderate (++) or strong (+++), comparing the slides side by side on a computer monitor to provide standardization from one sample to another. In addition to staining intensity, the distribution of positive CFD staining in tissue sections was analyzed.

### 2.6. Human cSCC Xenografts

Human cSCC xenografts were generated as previously described [26]. The metastatic cSCC cell line (UT-SCC-7) cells (5 × 10^6^) were injected subcutaneously in the back of severe combined immunodeficiency (SCID/SCID) female mice (CB17/Icr-*Prkdc^scid^*/IcrIcoCrl) (Charles River Laboratories). Xenograft tumors were harvested after 18 days and processed for IHC, as previously described [26,27,28,29], and labeled with FD antibody, as described above. The slides were digitally scanned using a Pannoramic 1000 Slide Scanner (3DHistech, Budapest, Hungary).

### 2.7. Targeted FD Inhibition and Cell Proliferation Assay

Specific small-molecule factor D inhibitor danicopan (ACH-4471, MedChemExpress (Monmouth Junction, NJ, USA)) was used to inhibit the activity of FD in cSCC cultures [18,41]. Cultured cSCC cells (UT-SCC-12A, UT-SCC-91, UT-SCC-59A, UT-SCC-105) were plated on 96-well plates (4000 cells/well) in the presence of 10% fetal calf serum. After overnight incubation, the small-molecule FD inhibitor danicopan (ACH-4471) was added to the wells in serum-free medium in different concentrations (0.1 µM, 1 µM and 10 µM). DMSO was added to control cultures as a vehicle control. Cell proliferation was determined with the IncuCyte S3 real-time cell imaging system (Essen Bioscience, Ann Arbor, MI, USA). Analysis of the results was performed using IncuCyte S3 software (Essen Bioscience, Ann Arbor, MI, USA). Relative confluence was measured as an index of cSCC cell proliferation.

### 2.8. Statistical Analysis

The significance of the differences between all sample groups except FD IHC staining intensity was calculated by either two-tailed Student’s *t*-test or Mann–Whitney U-test. Comparison of IHC staining intensity was performed with X^2^ test.

## 3. Results

### 3.1. Expression of FD Is Up-Regulated in cSCC Cells and Tumors

**Figure 1:**
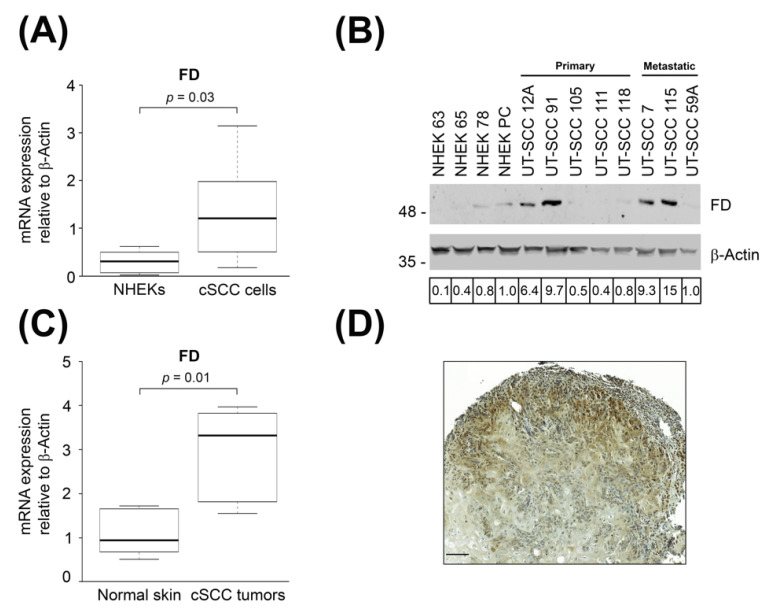
Expression of FD is up-regulated in cSCC tumor cells. (**A**): The levels of FD mRNA in normal human epidermal keratinocytes (NHEKs) (*n* = 6) and cSCC cell lines (*n* = 8) were quantitated by real-time qPCR (RT-qPCR) and corrected for β-Actin mRNA levels in the same samples. Horizontal bars demonstrate the mean values for each group. Statistical significance was determined by Mann–Whitney U test. (**B**): FD levels in conditioned media of NHEKs and primary and metastatic cSCC cell lines were determined by Western blot analysis under non-reducing conditions. β-Actin levels in the cell lysates were determined as the sample controls. Migration positions of molecular weight markers in kDa are shown on the left. Values indicated below the panels are relative to the levels in NHEK-PC cells (1.0). The uncropped immunoblot images can be found in Figure S1. (**C**): Levels of FD mRNA in normal skin (*n* = 6) and cSCC tumors (*n* = 6) were analyzed by RT-qPCR and corrected for the levels of β-Actin mRNA in the same samples. Horizontal bars represent the mean FD expression level for each group. Statistical significance was evaluated by a Mann–Whitney U test, (**D**): A xenograft tumor was generated by subcutaneous injection of human metastatic cSCC cell line (UT-SCC-7) into the back of SCID/SCID female mice. Xenograft was harvested after 18 days, and FD expression was analyzed with immunohistochemistry. Strong cytoplasmic staining for FD was detected in cSCC tumor cells in the invasive edge of the xenograft. Scale bar = 100 µm.

In our previous studies, the up-regulation of FD mRNA expression was detected in cSCC cell lines using oligonucleotide array (Affymetrix)-based gene expression profiling and RNA-seq based transcriptome profiling [25,26]. Herein, the mRNA levels of FD were quantified by RT-qPCR. The mean mRNA expression level of FD was significantly higher in cSCC cell lines as compared with NHEKs (Figure 1A). Levels of FD protein in the conditioned media of cSCC cell lines and NHEKs were determined by Western blot analysis. The results showed that the production of FD was markedly higher in four out of eight cSCC cell lines than in NHEKs (Figure 1B). Increased production of FD was observed in two primary cSCC cell lines (UT-SCC-12A and -91) and two metastatic cSCC cell lines (UT-SCC-7 and -115) (Figure 1B).

To determine the expression of FD in cSCC in vivo, RT-qPCR analysis was performed with RNA from cSCC tumors and normal skin. The results revealed significantly higher expression of FD mRNA in cSCC tumors compared to normal skin (Figure 1C). To further validate the expression of FD in vivo, xenograft tumors established with metastatic human cSCC cell line (UT-SCC-7) in (SCID/SCID) mice were analyzed with IHC. Cytoplasmic staining for FD was noted in the cSCC tumor cells in the invasive edges of the xenograft (Figure 1D). These results show that the expression of FD is specifically upregulated by tumor cells in cSCCs in vivo.

### 3.2. Expression of FD by Tumor Cells in cSCC In Vivo

**Figure 2:**
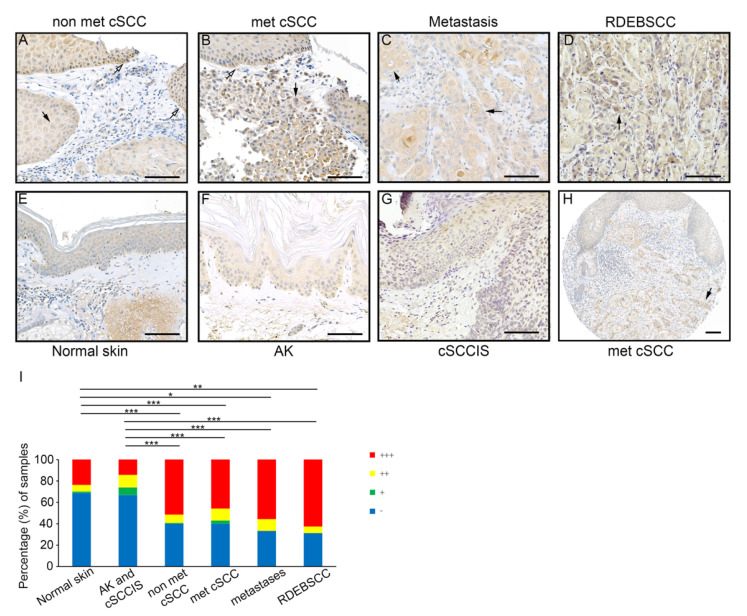
Expression of FD in cSCC tumor cells in vivo. (**A**–**G**): TMA sections of primary non-metastatic cSCC (*n* = 140), metastatic cSCC (*n* = 70), cSCC metastases (*n* = 9), RDEBSCC (*n* = 16), normal skin (*n* = 80), AK (*n* = 65) and cSCCIS (*n* = 61) were stained by immunohistochemistry with anti-FD antibody. Specific strong (+++) staining for FD was detected in tumor cells in the invasive edges (white arrows) and/or large more differentiated single cells (black arrows) of non-metastatic cSCC (**A**), metastatic cSCC (**B**), cSCC metastases (**C**) and in RDEBSCC (**D**). In normal skin (**E**), AK (**F**) and cSCCIS (**G**), the FD staining was weaker and showed a homogenous diffuse distribution through the whole epidermis. (**H**) Cytoplasmic FD staining was scored strong in metastatic cSCC cells of the tumor islands in dermal layer, whereas the adjacent epidermis showed weaker staining. (**I**) Semiquantitative analysis of FD staining in normal skin, AK, cSCCIS, non-metastatic cSCC, metastatic cSCC, cSCC metastases and RDEBSCC tissue sections. Cytoplasmic tumor cell–specific immunostaining for FD was scored as negative (−), weak (+), moderate (++) and strong (+++). * *p* < 0.05, ** *p* < 0.01, *** *p* < 0.001; X^2^ test. Scale bars = 100 µm.

Expression of FD in cSCC tumors in vivo was analyzed by IHC of TMAs containing tissue samples representing different stages of epidermal carcinogenesis including pre-malignant AK (*n* = 65), cSCCIS (*n* = 61), primary non-metastatic cSCC (*n* = 140), metastatic cSCC (*n* = 70) and cSCC metastases (*n* = 9) along with normal skin (*n*=80). Additionally, tissue samples of cSCCs from patients with recessive dystrophic epidermolysis bullosa (RDEB), a rare hereditary blistering disorder caused by germline mutations in the COL7A1 gene, which codes for type VII collagen, i.e., (RDEBSCC, *n* = 16), were examined as an example of an aggressive form of cSCC. Cytoplasmic labeling for FD was specifically strong (+++) in the tumor cells in invasive edges and in more differentiated cells of non-metastatic cSCC tumors (Figure 2A), metastatic cSCCs (Figure 2B) and cSCC metastases (Figure 2C). Furthermore, intense staining for FD was detected in the cytoplasm of tumor cells in RDEBSCC (Figure 2D). The labeling intensity for FD was weaker in normal skin (Figure 2E), AKs (Figure 2F) and cSCCISs (Figure 2G), and a homogenous pattern was observed in the whole epidermis area. Notably, in metastatic cSCC tissue sections in which strong cytoplasmic staining for FD was detected in the invasive tumor islands, the intensity of staining in the adjacent epidermis was clearly weaker (Figure 2H). The staining intensity of cytoplasmic FD was semiquantitatively scored as negative (−), weak (+), moderate (++) or strong (+++). The analysis showed that the majority of cSCC samples were scored strong regardless of their metastatic status (Figure 2I). Interestingly, FD staining was as intense in metastatic cSCCs, cSCC metastases or aggressive RDEBSCCs as in primary non-metastatic cSCC tumors (Figure 2I). The intensity of staining in normal skin, AKs and cSCCISs was predominantly scored weak (Figure 2I). These results indicate that the expression of FD by tumor cells is upregulated during the progression of cSCC to the invasive stage.

### 3.3. Expression of FD in cSCC Cells Is Up-Regulated by IFN-γ and IL-1β

**Figure 3:**
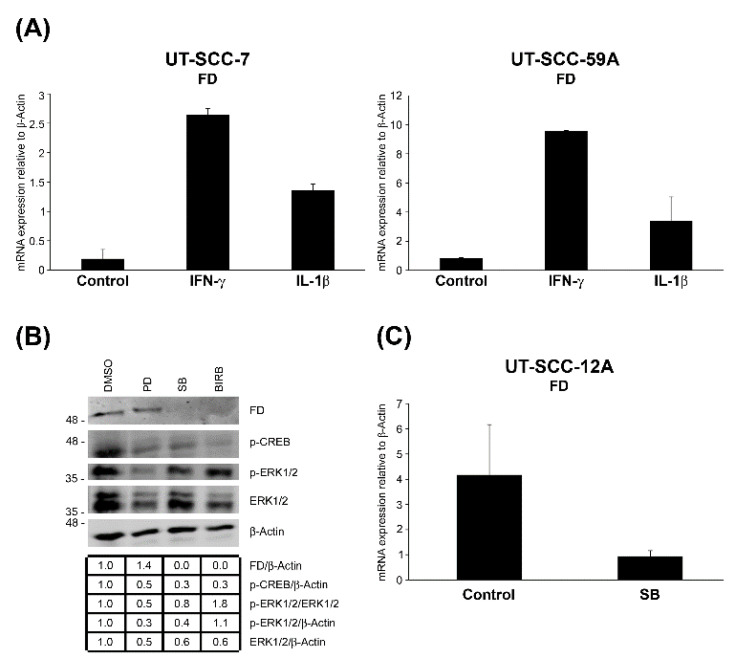
Up-regulation of FD expression in cSCC cells by interferon-γ and interleukin-1β, and p38 MAPK signaling pathway. (**A**), cSCC cells in culture were treated with interferon-γ (IFN-γ; 100 IU/mL) or interleukin-1β (IL-1β; 10 ng/mL) for 24 h. The expression levels of FD mRNA were analyzed by RT-qPCR, and the levels were corrected for β-Actin mRNA levels in the same samples. Mean ± S.D. is shown. (**B**), cSCC cells (UT-SCC-12A) were treated with MEK1/2 inhibitor (PD98059; 30 µM) or p38 inhibitor specific for p38α/β (SB203580; 10 ÌM) or the inhibitor of all p38 isoforms α, β, γ and δ (BIRB796; 10 μM) for 24 h. The conditioned media were analyzed for levels of FD by Western blotting. Cell lysates were analyzed for levels of phosphorylated CREB (p-CREB), phosphorylated ERK1/2 (p-ERK1/2) and ERK1/2 to verify the proper effects of SB203580, BIRB and PD98059, respectively. β-actin was used as a sample and loading control. Migration of molecular weight markers in kDa is shown on the left. The uncropped immunoblot images can be found in Figure S2. (**C**), The levels of FD mRNA in cSCC cells in culture (UT-SCC-12A) treated with SB203580 were determined using RT-qPCR and corrected for β-Actin mRNA levels in the same samples. Mean ± S.D. is shown.

To elucidate the regulation of FD expression in cSCC cells, cultures of two metastatic cSCC cell lines (UT-SCC-7 and -UT-SCC-59A) were treated with inflammatory cytokines interferon-γ (IFN-γ) and interleukin-1β (IL-1β) for 24 h. The FD mRNA levels were up-regulated by IFN-γ and IL-1β in both cSCC cell lines (Figure 3A).

### 3.4. FD Expression by cSCC Cells Is Regulated by p38 MAPK Pathway

To further investigate the regulation of FD expression in cSCC cells, they were treated with MAPK/ERK kinase 1/2 (MEK1/2) inhibitor (PD98059), p38 inhibitor selective for p38α and p38β (SB203580) or the inhibitor of all p38 isoforms α, β, γ and δ (BIRB796) for 24 h. Basal production of FD protein in cSCC cells was significantly down-regulated by SB203580 and BIRB796 compared to untreated control cultures, whereas treatment with PD98059 had no effect on FD production (Figure 3B). A marked decrease in ERK1/2 activation was observed as a verification for the efficiency of PD98059, but this had no effect on the basal production of FD (Figure 3B). At the mRNA level, a significant reduction in FD expression was noted in cSCC cells treated with SB203580 as compared to untreated control cells (Figure 3C). Considering the lack of p38β expression in cSCC cells [42], these findings imply that p38α MAPK is involved in the regulation of the basal FD expression in cSCC cells.

### 3.5. Expression of FD in Tumorigenic Ha-ras-Transformed HaCaT cells

**Figure 4:**
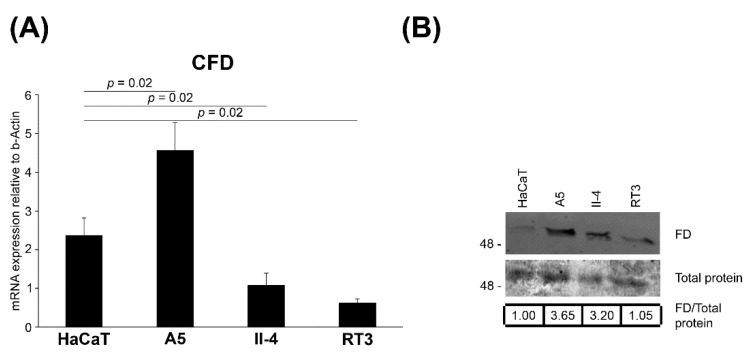
Regulation of FD expression in HaCaT and tumorigenic Ha-ras-Transformed HaCaT cell lines in culture. (**A**), FD mRNA levels were quantified by RT-qPCR in HaCaT cells, a nontumorigenic epidermal keratinocyte-derived cell line and in three Ha-ras-transformed HaCaT cell lines (A5, II-4 and RT3). A5 cells form benign, II-4 cells invasive malignant and RT3 cells metastatic tumors in vivo. The mRNA levels were corrected for levels of β-Actin mRNA in the same samples. Mean ± S.D. are shown; two-tailed *t*-test. (**B**), The expression of FD protein in conditioned media of HaCaT and Ha-ras-transformed HaCaT cell lines (A5, II-4 and RT3) was determined by Western blot analysis under non-reducing conditions. Equal total protein loading was controlled by Ponceau (0.2%) staining. Migration of molecular weight markers in kDa is shown on the left. Values shown below the Western blots are relative to the levels in HaCaT cells (1.00), The uncropped immunoblot images can be found in Figure S3.

To examine the significance of FD in epidermal carcinogenesis, FD expression was investigated in an immortalized nontumorigenic cell line (HaCaT) derived from human epidermal keratinocytes as well as three Ha-ras-transformed HaCaT cell lines (A5, II-4 and RT3), which are representative in vitro models for the progressive stages of cSCC tumor. Particularly, A5 cells form benign, II-4 cells low-grade malignant (primary invasive), and RT3 cells high-grade malignant (metastatic) tumors in vivo [35,36]. In addition, II-4 and RT3 cells display markedly higher basal ERK1/2 activation than A5 cells [43]. The expression of FD mRNA was notably lower in nontumorigenic HaCaT cells, lacking functional p53, compared with benign Ha-ras-transformed A5 cells (Figure 4A). Interestingly, low-grade malignant II-4 cells and high-grade malignant RT3 cells expressed significantly lower levels of FD mRNA in comparison to the benign Ha-ras-transformed A5 cells (Figure 4A). Similarly, the production of FD protein was lower in II-4 and RT3 cells than in A5 cells (Figure 4B). These results provide evidence that basal FD expression is upregulated at an early stage of epidermal carcinogenesis and is further down-regulated by potent constitutive activation of ERK1/2.

### 3.6. Targeted Inhibition of FD Inhibits Proliferation of cSCC Cells via Blockade of ERK1/2 Activation

**Figure 5:**
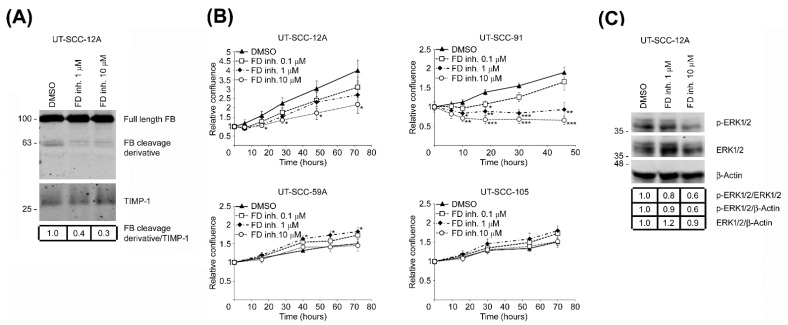
Targeted inhibition of FD suppresses the proliferation of cSCC cells through blockade of ERK1/2 activation. (**A**), cSCC cell cultures (UT-SCC-12A) were treated with a small-molecule factor D inhibitor (danicopan; ACH-4471) or DMSO as vehicle control for 24 h. The levels of complement factor B (FB) and its 60 kDa cleavage derivative in conditioned media of cSCC cells were determined by Western blot analysis. TIMP-1 was used as a loading control. Migration positions of molecular weight markers in kDa are shown on the left, The uncropped immunoblot images can be found in Figure S4. (**B**), cSCC cells with high basal FD expression (UT-SCC-12A and -91) and with low basal FD expression (UT-SCC-59A and -105) were treated with small-molecule FD inhibitor (danicopan; ACH-4471) or DMSO as vehicle control. Targeted inhibition of FD significantly suppressed cell proliferation, specifically in cSCC cell lines with high expression of FD protein (UT-SCC-12A and-91), whereas no similar effect was observed on cSCC cells with low FD protein expression (UT-SCC-59A and-105). Results of cell proliferation assays after targeted FD inhibition using IncuCyte S3 real-time cell imaging system are shown. * *p* < 0.05, ** *p* < 0.01, *** *p* < 0.001; two-tailed *t*-test. (**C**), Levels of phosphorylated ERK1/2 (p-ERK1/2) and ERK1/2 in small-molecule factor D inhibitor (danicopan; ACH-4471) or DMSO vehicle-treated cSCC cell lysates were determined by Western blot analysis 24 h following targeted FD inhibition. β-Actin was used as a loading control. Migration positions of molecular weight markers in kDa are shown on the left, The uncropped immunoblot images can be found in Figure S5.

To elucidate the mechanistic role of FD in cSCC cell proliferation, cSCC cells were treated with different concentrations of the small-molecule FD inhibitor danicopan (ACH-4471) or DMSO as a vehicle control, and the proliferation of cells was determined. Western blot analysis of conditioned media of danicopan-treated cSCC cells showed considerably reduced levels of the 60 kDa FB cleavage derivative following targeted inhibition of FD (Figure 5A). A significant dose-dependent decrease in proliferation was discovered in danicopan-treated cSCC cells (UT-SCC-12A and -91) compared to DMSO-treated control cultures (Figure 5B). In contrast, danicopan had no inhibitory effect on the proliferation of two other cSCC cell lines with low FD expression (UT-SCC-59A and -105) (Figure 5B). Inhibition of ERK1/2 activation was detected after targeted FD inhibition (Figure 5C).

## 4. Discussion

In the present study, we have examined the role of FD in keratinocyte-derived carcinoma, cSCC. Previous studies have shown the expression of FD by adipocytes, macrophages and liver cells and in some cancer cells [17,18], but the expression in epidermal keratinocytes or in keratinocyte carcinomas is not known. Our results show increased expression of FD mRNA levels in cSCC cells in vitro and in cSCC tumors in vivo compared to NHEKs and normal skin, respectively. In addition, elevated production of FD protein by four out of eight cSCC cell lines was noted by Western blotting, and the levels of FD protein were highest in two metastatic cSCC cell lines (UT-SCC-115 and-7) and in two primary cSCC cell lines (UT-SCC-91 and-12A). Marked expression of FD was also noted in tumor cells in the invasive margin in xenografts established with metastatic cSCC cell line UT-SCC-7. These results show that FD expression is specifically upregulated by tumor cells in cSCCs in vivo.

Analysis of TMAs consisting of a large panel of AK, cSCCIS, non-metastatic cSCC, metastatic cSCC, cSCC metastases, RDEBSCC and normal skin by IHC revealed specific cytoplasmic staining for FD in tumor cells in cSCCs in vivo. Staining for FD was significantly stronger in each of the cSCC tumors mentioned above compared to normal skin, AK, and cSCCIS. Interestingly, the intensity of FD staining was not dependent on the metastatic status of the primary tumor. Strong FD expression was specifically localized to invasive edges of the tumor and to more differentiated cells in cSCC, whereas weak FD staining with a diffuse homogenous pattern of distribution was detected in normal epidermis, AK and cSCCIS. In metastatic cSCC tissue sections with strong staining for FD localized to the invasive tumor islands, the intensity of staining in the adjacent epidermis was distinctly weaker. Altogether these results indicate that FD is specifically induced in cSCC tumor cell margin and in invasive cSCC cells and suggest a role for FD in the progression of cSCC to the invasive stage.

A typical histological characteristic of cSCC is the influx of inflammatory cells in the tumor microenvironment [9,11,44]. Cytokines secreted by these inflammatory cells induce the production of invasion-associated proteinases and consequently promote progression and invasion of cSCC [10]. In addition, the local immune reaction to tumor antigens is regulated by tumor cell-released cytokines [45,46]. In the current study, we found out that FD expression by cSCC cells is augmented by two proinflammatory cytokines (i.e., IFN-γ and IL-1β). Moreover, the basal expression of FD in cSCC cells was regulated by the p38 MAPK signaling pathway, in particular by p38α MAPK, which supports the observations of previous studies highlighting the role of p38 MAPKs in cSCC progression [42,47,48].

The mutation of the *TP53* gene and inactivation of the tumor suppressor p53 in epidermal keratinocytes is an early event in epidermal carcinogenesis, which results in the accumulation of oncogenic mutations required for the progression of AK to invasive cSCC [49]. In order to assess the role of FD as a potential molecular marker for cutaneous carcinogenesis, an in vitro model of different stages of keratinocyte carcinogenesis was developed and tested [34,35,36]. The basal expression level of FD at mRNA and protein level was significantly higher in the benign tumorigenic Ha-ras-transformed HaCaT cell line A5 than in the immortalized nontumorigenic cell line (HaCaT). Interestingly, the expression of FD was also higher in the benign tumorigenic cell line A5 than in the primary invasive tumorigenic cell line II-4 and metastatic tumorigenic cell line RT3, which both show high basal activation of ERK1/2 [43]. These observations reveal that the expression mechanism of FD in cSCC cells is complex and not exclusively dependent on p53 inactivation or ras-transformation. Additionally, the results imply that FD might be a putative biomarker for the early stages of cSCC progression.

To elucidate the functional role of FD in cSCC cell proliferation, a small-molecule factor D inhibitor (danicopan; ACH-4471; ALXN2040) was used [18,41]. The targeted inhibition of FD expression in cSCC cell cultures with high FD expression resulted in potent suppression of cSCC cell proliferation and ERK1/2 activation. Interestingly, danicopan had no inhibitory effect on the proliferation of the cSCC cell lines with low FD expression. Additionally, cleavage of FB was shown to be potently reduced after FD inhibition, according to the mechanistic role of FD on FB in the AP of the complement system. In summary, these findings provide evidence for the role of FD in cSCC progression through regulation of ERK1/2 signaling pathway and validate the sensitivity and specificity of danicopan as a potential targeted therapy of advanced cSCC expressing a high level of FD.

Previous studies have demonstrated the production of FD by cancer cell lines, such as gastric cancer-derived cell lines and astroglioma cell line U105-MG [50,51]. Furthermore, FD secreted by mammary adipose tissue has been shown to promote the proliferation and growth of human breast cancer and boost the stem cell-like properties of the malignancy [52]. In a recent analysis of the TCGA database, elevated expression of FD was noted in the majority of the cancers associated with high expression of FB [23]. Interestingly, high FD expression was found to be associated with poor survival in adrenocortical carcinoma, thyroid carcinoma, uveal melanoma, lower-grade glioma and glioblastoma [23]. Together with the high local expression of C3, this suggests that complement could be activated via the alternative pathway in the tumor microenvironment. These observations are interesting in light of our previous findings showing high tumor cell-specific expression of C3 and FB by cSCC cells in culture and in vivo [27]. In addition, recent observations show that C3 promotes cSCC progression independently of C5, indicating that activation of C3 via AP plays an important role in cSCC progression [53].

Increased understanding of the interplay between cancer cells and the immune system has revealed the central role of immune response in the development and growth of malignant tumors [54]. As the focal pillar of innate immunity, the complement cascade plays a significant role in the progression of cancers along with inflammatory diseases [11,20,21,22,23,54]. At present, several complement-targeted therapeutic compounds are in clinical trials and in preclinical development [11]. The major focus of complement-targeted therapies has been inflammatory disorders [11]. Clinical trials with three oral small-molecule FD inhibitors for the treatment of inflammatory conditions are underway. Specifically, danicopan (ACH-4471 or ALXN2040) is being examined in targeted therapy of paroxysmal nocturnal hemoglobinuria (PNH) (phases 2 and 3) [55,56,57], C3 glomerulopathy (C3G) and immune complex membranoproliferative glomerulonephritis (IC-MPGN) (phase 2) [58], geographic atrophy (GA) secondary to age-related macular degeneration (AMD) (phase 2) [59] and COVID-19 (ACTIV-5 / Big Effect Trial (BET-C), phase 2) [60]. In addition, phase 2 clinical trials with small-molecule FD inhibitors vemircopan (ACH-5228 or ALXN2050) and BCX9930 for PNH are ongoing [61,62].

Recent studies have revealed the importance of local or autocrine production and activation of complement components compared to systemic liver-derived complement synthesis [25,26,27,28,29,30]. However, only anaphylatoxin receptors C5aR1 and C3aR, as well as anaphylatoxin C5a, have been targeted in anti-cancer therapy [11]. IPH-5401 is an anti-C5aR1 antibody presently in phase 1 clinical trial for the treatment of selected advanced solid tumors in combination with anti-PD-L1 durvalumab (STELLAR-001 study) [11,63]. AON-D21 (formerly known as NOX-D21) is a Pegylated C5a-neutralizing L-configured aptamer in the first-in-human (phase 1) clinical trial on healthy males [11,64]. In this respect, the results of the present study identify FD as a potential therapeutic target in cSCC and warrant further studies to evaluate the small-molecule FD inhibitor danicopan in targeted therapy of advanced cSCC and other cancers expressing high levels of FD.

## 5. Conclusions

**Figure 6:**
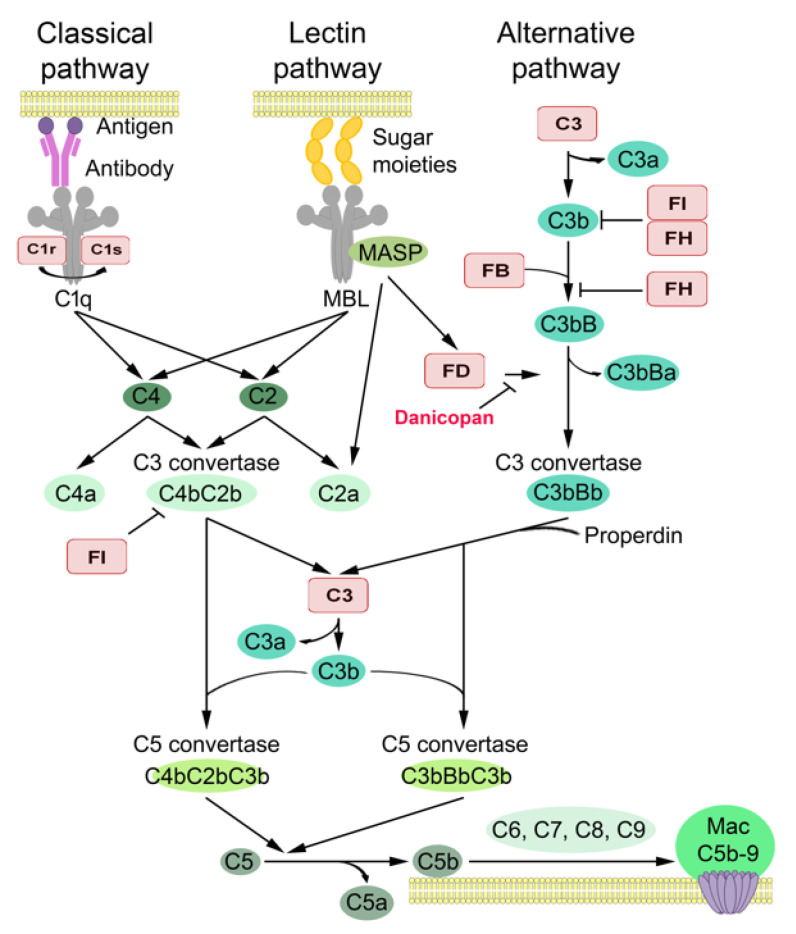
Complement components in cSCC. The complement may be activated via the classical, lectin or alternative pathway. The complement components and inhibitors shown to promote the progression of cSCC are highlighted in red. C3, FB and FI promote cSCC tumor growth in vivo [26,27]. C1r and C1s promote tumor growth and vascularization of cSCC xenograft tumors in vivo [28]. C3, FB, C1r, C1s, FI and FH increase cSCC cell viability, proliferation and migration in culture [25,26,27,28]. C1r and FI promote invasion of cSCC cells; C1r by increasing expression of MMP-13, MMP-1, MMP-10 and MMP-12 and FI by increasing expression of MMP-13 and MMP-2 [29,30]. FD regulates the proliferation of cSCC cells, and the specific inhibitor of FD (danicopan; ACH-4471) inhibits the proliferation of cSCC cells in a dose-dependent manner. Modified from [11].

The results of the present study show that the alternative complement system component FD is specifically overexpressed by tumor cells in cSCC in vivo, implying that this complement component plays a role in cSCC development and progression. Additionally, our findings demonstrate that FD promotes the proliferation of cSCC cells via the regulation of the ERK1/2 signaling pathway. These results identify FD as a putative tumor cell-associated biomarker and therapeutic target of cSCC and, for the first time, introduce the small-molecule FD inhibitor danicopan as a highly sensitive and specific drug in precision cancer therapy (Figure 6). Together with our previous studies showing the role of FB, C3, FI and FH in AP, and C1r and C1s in the classical pathway in cSCC progression [25,26,27,28,29,30], these results provide further evidence for the role of specific complement components as biomarkers and potential therapeutic targets in cSCC (Figure 6).

## Data Availability

The data presented in this study are available on request from the corresponding author.

## Supplementary Materials

The following are available online at https://www.mdpi.com/article/10.3390/cancers14020305/s1, Figure S1: Expression of FD is up-regulated in cSCC tumor cells. B: FD levels in conditioned media of NHEKs and primary and metastatic cSCC cell lines were determined by Western blot analysis under nonreducing conditions. β-Actin levels in the cell lysates were determined as the sample controls. Migration positions of molecular weight markers in kDa are shown on the left. Figure S2: Up-regulation of FD expression in cSCC cells by IFN- γ and IL-1β, and p38 MAPK signaling pathway. B: cSCC cells (UT-SCC-12A) were treated with MEK1/2 inhibitor (PD98059; 30 µM) or p38 inhibitor specific for p38α/β (SB203580; 10 μM) or the inhibitor of all p38 isoforms α, β, γ and δ (BIRB796; 10 µM) for 24 hours. The conditioned media were analyzed for FD levels by Western blot analysis. Cell lysates were analyzed for levels of phosphorylated CREB (p-CREB), phosphorylated ERK1/2 (p-ERK1/2) and ERK1/2 to verify the proper effects of SB203580, BIRB, and PD98059, respectively. β-actin was used as a sample and loading control. Migration positions of molecular weight markers in kDa are shown on the left. Figure S3: Regulation of FD expression in HaCaT and tumorigenic Ha-ras–Transformed HaCaT cell lines in culture. B: The expression of FD protein in conditioned media of HaCaT and Ha-ras-transformed HaCaT cell lines (A5, II-4, and RT3) was determined by Western blot analysis under nonreducing conditions. Equal total protein loading was controlled by Ponceau (0.2%) staining. Migration positions of molecular weight markers in kDa are shown on the left. Figure S4: Targeted inhibition of FD suppresses proliferation of cSCC cells through blockade of ERK1/2 activation. A: cSCC cell cultures (UT-SCC-12A) were treated with small-molecule factor D inhibitor (Danicopan; ACH-4471) or DMSO as vehicle control for 24 h. The levels of complement factor B (FB) and its 60 kDa cleavage derivative in conditioned media of cSCC cells were determined by Western blot analysis. TIMP-1 was used as a loading control. Migration positions of molecular weight markers in kDa are shown on the left. Figure S5: Targeted inhibition of FD suppresses proliferation of cSCC cells through blockade of ERK1/2 activation. C: Levels of phosphorylated ERK1/2 (p-ERK1/2) and ERK1/2 in small-molecule factor D inhibitor (Danicopan; ACH-4471) or DMSO vehicle treated cSCC cell lysates were determined by Western blot analysis 24 h following targeted FD inhibition. β-Actin was used as loading control. Migration positions of molecular weight markers in kDa are shown on the left.
