# Supplementary material for: Complement Factor D Is a Novel Biomarker and Putative Therapeutic Target in Cutaneous Squamous Cell Carcinoma
Source: Cancers (Basel). 2022 Jan 8;14(2):305. doi: 10.3390/cancers14020305 (PMC8773783; doi:10.3390/cancers14020305)
Supplement: Supplementary file 1 [file cancers-14-00305-s001.zip › cancers-1516781-supplementary.pdf]

Supplementary Materials

Complement Factor D Is a Novel Biomarker and Putative Therapeutic Target in Cutaneous Squamous Cell Carcinoma

Pegah Rahmati Nezhad, Pilvi Riihilä, Jaakko S. Knuutila, Kristina Viiklepp, Sirkku Peltonen, Markku Kallajoki, Seppo Meri, Liisa Nissinen and Veli-Matti Kähäri

Original uncropped Western blots for Figures 1B, 3B, 4B, 5A, 5C

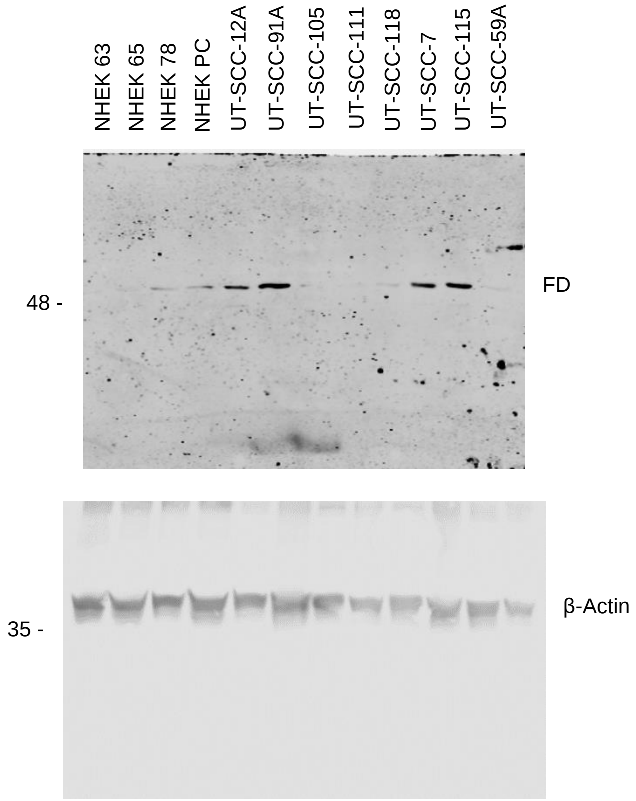

Figure S1. Expression of FD is up-regulated in cSCC tumor cells. B: FD levels in conditioned media of NHEKs and primary and metastatic cSCC cell lines were determined by Western blot analysis under nonreducing conditions. β-Actin levels in the cell lysates were determined as the sample controls. Migration positions of molecular weight markers in kDa are shown on the left.

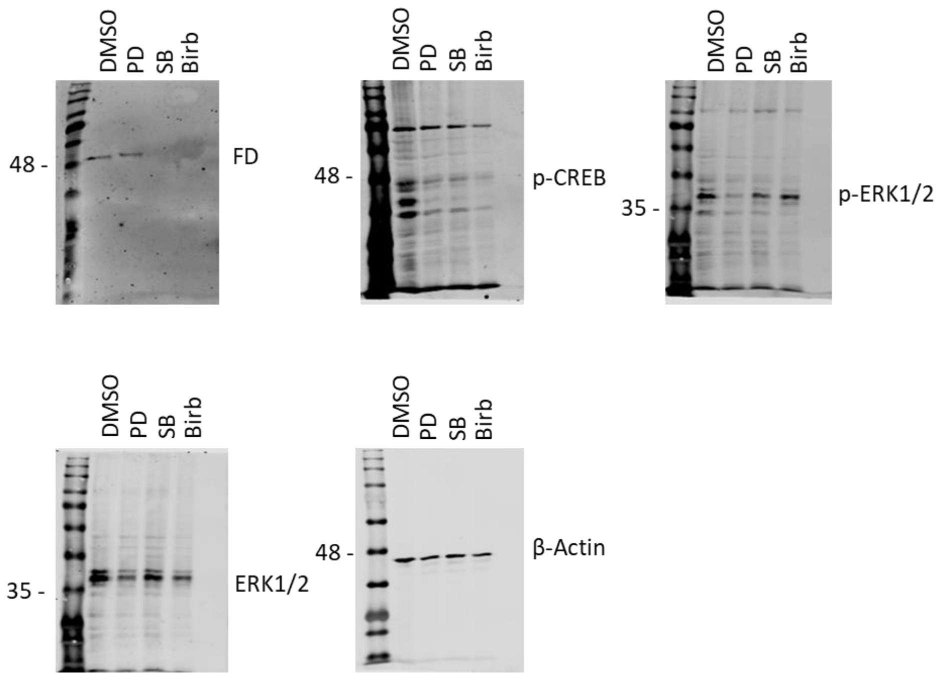

Figure S2. Up-regulation of FD expression in cSCC cells by IFN- γ and IL-1β, and p38 MAPK signaling pathway. B: cSCC cells (UT-SCC-12A) were treated with MEK1/2 inhibitor (PD98059; 30 μM) or p38 inhibitor specific for p38α/β (SB203580; 10 μM) or the inhibitor of all p38 isoforms α, β, γ and δ (BIRB796; 10 μM) for 24 hours. The conditioned media were analyzed for FD levels by Western blot analysis. Cell lysates were analyzed for levels of phosphorylated CREB (p-CREB), phosphorylated ERK1/2 (p-ERK1/2) and ERK1/2 to verify the proper effects of SB203580, BIRB, and PD98059, respectively. β-actin was used as a sample and loading control. Migration positions of molecular weight markers in kDa are shown on the left.

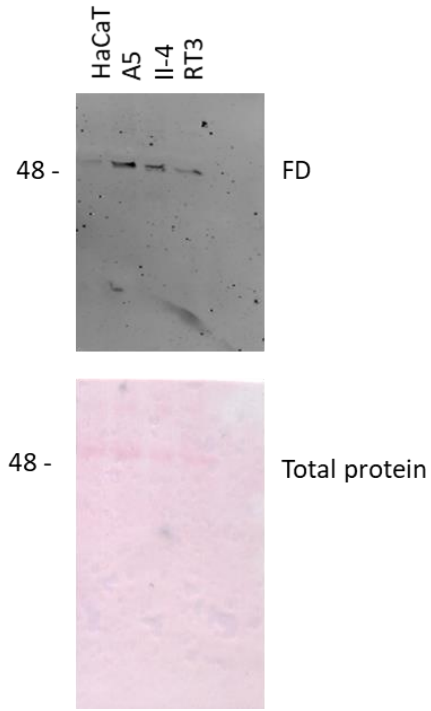

Figure S3. Regulation of FD expression in HaCaT and tumorigenic Ha-ras–Transformed HaCaT cell lines in culture. B: The expression of FD protein in conditioned media of HaCaT and Ha-ras-transformed HaCaT cell lines (A5, II-4, and RT3) was determined by Western blot analysis under nonreducing conditions. Equal total protein loading was controlled by Ponceau (0.2%) staining. Migration positions of molecular weight markers in kDa are shown on the left.

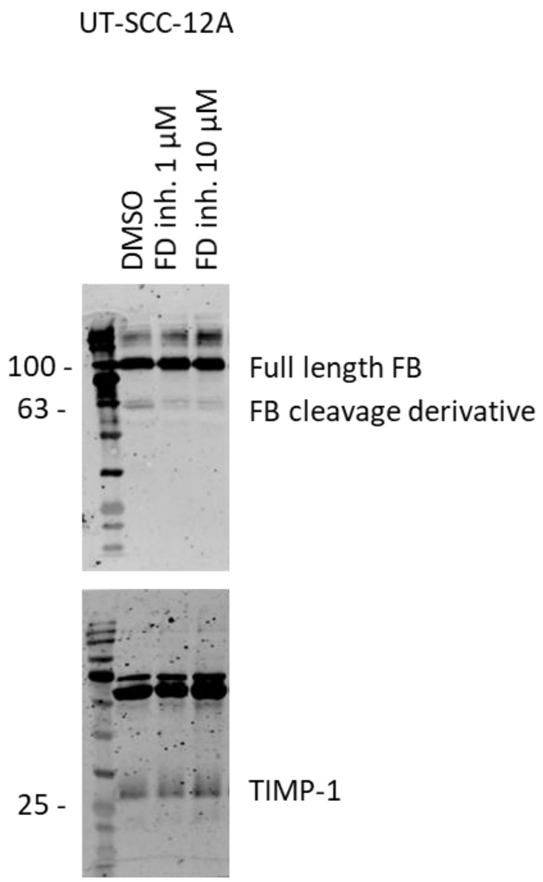

Figure S4. Targeted inhibition of FD suppresses proliferation of cSCC cells through blockade of ERK1/2 activation. A: cSCC cell cultures (UT-SCC-12A) were treated with small-molecule factor D inhibitor (Danicopan; ACH-4471) or DMSO as vehicle control for 24 h. The levels of complement factor B (FB) and its 60 kDa cleavage derivative in conditioned media of cSCC cells were determined by Western blot analysis. TIMP-1 was used as a loading control. Migration positions of molecular weight markers in kDa are shown on the left.

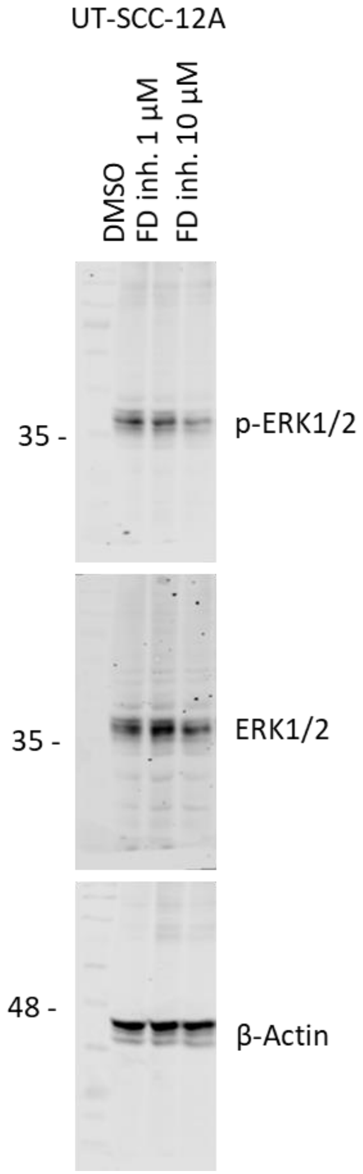

Figure S5. Targeted inhibition of FD suppresses proliferation of cSCC cells through blockade of ERK1/2 activation. C: Levels of phosphorylated ERK1/2 (p-ERK1/2) and ERK1/2 in small-molecule factor D inhibitor (Danicopan; ACH-4471) or DMSO vehicle treated cSCC cell lysates were determined by Western blot analysis 24 h following targeted FD inhibition. β-Actin was used as loading control. Migration positions of molecular weight markers in kDa are shown on the left.
